# Optical backaction-evading measurement of a mechanical oscillator

**DOI:** 10.1038/s41467-019-10024-3

**Published:** 2019-05-07

**Authors:** Itay Shomroni, Liu Qiu, Daniel Malz, Andreas Nunnenkamp, Tobias J. Kippenberg

**Affiliations:** 1 Institute of Physics, École Polytechnique Fédérale de Lausanne, Station 3, CH-1015 Lausanne, Switzerland; 2 Max-Planck-Institut für Quantenoptik, Hans-Kopfermann-Strasse 1, 85741 Garching, Germany; 3 Cavendish Laboratory, University of Cambridge, Cambridge, CB3 0HE UK

**Keywords:** Single photons and quantum effects, Optomechanics, Quantum metrology

## Abstract

Quantum mechanics imposes a limit on the precision of a continuous position measurement of a harmonic oscillator, due to backaction arising from quantum fluctuations in the measurement field. This standard quantum limit can be surpassed by monitoring only one of the two non-commuting quadratures of the motion, known as backaction-evading measurement. This technique has not been implemented using optical interferometers to date. Here we demonstrate, in a cavity optomechanical system operating in the optical domain, a continuous two-tone backaction-evading measurement of a localized gigahertz-frequency mechanical mode of a photonic-crystal nanobeam cryogenically and optomechanically cooled close to the ground state. Employing quantum-limited optical heterodyne detection, we explicitly show the transition from conventional to backaction-evading measurement. We observe up to 0.67 dB (14%) reduction of total measurement noise, thereby demonstrating the viability of backaction-evading measurements in nanomechanical resonators for optical ultrasensitive measurements of motion and force.

## Abstract (web summary)

Measurements of motion that avoid quantum backaction, with the potential to surpass the standard quantum limit, have so far been demonstrated using microwave radiation. Here, Shomroni, Qiu et al. demonstrate a backaction-evading measurement of the motion of a nanomechanical beam using laser light.

## Introduction

In a continuous measurement of the position $\hat x$ of a harmonic oscillator, quantum backaction (QBA) of the measuring probe on the momentum $\hat p$ ultimately limits the attainable precision^1,2^, restricting ultrasensitive measurements of force or motion. For an interferometric position measurement, in which a mechanical oscillator is parametrically coupled to a cavity, the trade-off arising from measurement imprecision (i.e., detector shot noise) and QBA force noise on the mechanical oscillator, dictates a minimum added noise equivalent to the oscillator’s zero-point fluctuations, $x_{\mathrm{zpf}} = \sqrt {\hbar {\mathrm{/}}2m\Omega_{\mathrm{m}}}$, referred to as the standard quantum limit (SQL), originally studied in the context of gravitational wave detection^1,3^ (here *m* is the mass and Ω_m_ the angular frequency of the mechanical oscillator). Recent advances in the field of cavity optomechanics^4^, which utilizes a nano- or micromechanical oscillator coupled to an optical or superconducting microwave cavity, have allowed reaching the regime where the QBA arising from radiation pressure quantum fluctuations becomes relevant. In particular, imprecision noise far below that at the SQL has been obtained^5,6^, thus entering the QBA-dominated regime; QBA has been observed^7–9^ and sensitivities approaching the SQL have been demonstrated^9–12^.

Quantum non-demolition (QND) techniques, first proposed by Thorne, Braginsky, and colleagues^13–15^, allow beating the SQL by minimizing or evading the effects of QBA. One technique to surpass the SQL, applicable to measurements far from the mechanical resonance frequency Ω_m_, utilizes quantum correlations in the probe (due to ponderomotive squeezing^16–19^), known as “variational readout”^20,21^. This technique has recently been demonstrated in a cryogenic micromechanical oscillator coupled to an optical cavity^22^ and in a room-temperature nano-optomechanical system for quantum-enhanced force measurements^23^. Another possibility is utilizing squeezed light, a technique applied to gravitational wave detectors^21,24,25^. More recent schemes include measurements of the collective motion in a hybrid system composed either of two mechanical oscillators (as demonstrated for an electromechanical system^26,27^) or a mechanical and a “negative mass” oscillator (demonstrated using an atomic ensemble^28,29^).

Another type of QND measurement, backaction-evading (BAE) measurements introduced by Thorne et al.^13^, allow avoiding QBA entirely by measuring only one of the two slowly varying amplitude and phase quadratures $\hat X$ and $\hat Y$, defined by $\hat x(t) \equiv \sqrt 2 x_{{\mathrm{zpf}}}[\hat X(t)\cos\Omega _{\mathrm{m}}t + \hat Y(t)\sin\Omega _{\mathrm{m}}t]$, which constitute QND observables. Unlike $\hat x$ and $\hat p$, the conjugate observables $\hat X$ and $\hat Y$ are decoupled from each other during free dynamic evolution. By exclusively measuring $\hat X$, e.g., all QBA is diverted to $\hat Y$ and is completely absent from the measurement record. By increasing coupling to the system (probe power), one can then arbitrarily reduce the imprecision noise, allowing in principle unlimited sensitivity in the measurement of one quadrature. In a cavity optomechanical system, such BAE measurement is possible by amplitude-modulating a cavity-resonant probe at frequency Ω_m_^14,15,30^, equivalent to two-tone probing on the upper and lower mechanical sidebands of the cavity. It has been pointed out^22^ that single-quadrature measurement may also be realized using synodyne detection^31^. Two-tone BAE is applicable in the well-resolved sideband regime $\Omega _{\mathrm{m}} \gg \kappa$, where $\kappa$ is the cavity linewidth. In the opposite regime of a fast cavity $\kappa \gg \Omega _{\mathrm{m}}$, one must resort to stroboscopic QND measurements, requiring interaction times $\ll \Omega _{\mathrm{m}}^{ - 1}$ (refs ^1,14^).

To date, such two-tone BAE measurements have exclusively been demonstrated in microwave optomechanical systems^32,33^, where they have also been utilized to perform tomography of states produced by schemes that produce reservoir-engineered squeezed^34–38^ and entangled^39,40^ mechanical states. Yet, in all these experiments, noise resulting from the use of a microwave amplifiers at elevated temperatures resulted in substantially decreased efficiency and hindered beating the SQL^33^. In addition, thermal noise at microwave frequencies can be non-negligible even at cryogenic temperatures and requires careful calibration^41^. In contrast, optical homodyne or heterodyne detection is quantum-limited and light is effectively a zero-temperature bath, allowing self-calibrated measurements of motion^42–44^. Optomechanical systems using laser light have demonstrated quantum effects up to room temperature^23,45^. Despite advances in operating in the QBA-dominated regime in cavity optomechanics, BAE measurements in the optical domain have not been reported.

Here we demonstrate a two-tone BAE measurement in the optical domain of an oscillator in a thermal state, using quantum-limited balanced heterodyne detection (BHD). We observe explicitly the reduction of thermomechanical noise due to cancellation of QBA from the measuring probes. We analyze the effect of extraneous heating due to optical absorption, which limits the attainable sensitivity in the present sample.

## Results

### Theoretical model

**Fig. 1:**
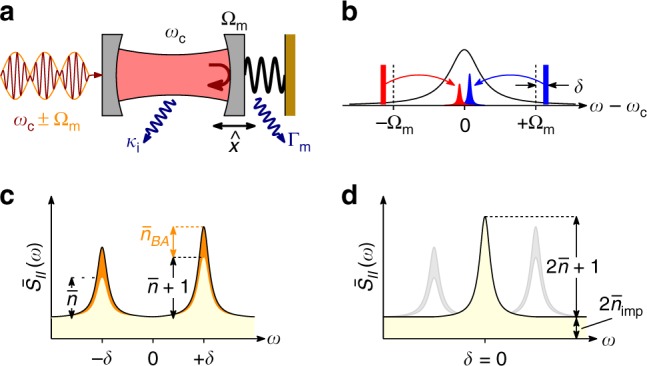
Backaction-evading measurement. **a** Illustration of a cavity optomechanical system. Light in a cavity with optical resonance *ω*_c_ and full linewidth *κ* (of which *κ*_i_ are intrinsic losses) is coupled to the position $\hat x$ of a mechanical oscillator that has frequency Ω_m_ and linewidth Γ_m_. In a backaction-evading (BAE) measurement, the probe is amplitude-modulated at the mechanical frequency Ω_m_, coupling to the quadrature $\hat X$. **b** Frequency space configuration slightly detuned from BAE measurement, where the probe is modulated at Ω_m_ + *δ*. **c** Resulting power spectral density of the cavity output field for an oscillator in a thermal state (mean occupation $\bar n$), showing the asymmetric Stokes and anti-Stokes scattered sidebands, plus the heating due to QBA. **d** When tuning to the BAE scheme *δ* = 0, the two sidebands coalesce and the QBA is cancelled, see Eq. (1). The remaining imprecision noise $\overline n _{{\mathrm{imp}}}$ can be arbitrarily reduced by increasing probe power

We first consider theoretically the scenario depicted in Fig. 1b–d, in which a cavity optomechanical system is interrogated with two tones detuned by $\pm (\Omega _{\mathrm{m}} + \delta )$ from the cavity resonance and the two sidebands are detected using BHD. The mechanical oscillator is in a thermal state with mean occupation $\bar n$. In the case of a quantum-limited laser, in the well-resolved sideband regime $\Omega _{\mathrm{m}} \gg \kappa$, and within the rotating-wave approximation (RWA), the measured photocurrent power spectral density (PSD) is given by (Methods section)

1 $$\begin{array}{l}\bar S_{II}(\omega ) = 1 + \eta \Gamma _{{\mathrm{eff}}}^2{\cal{C}}\left[ {\bar n\left| {\chi _{\mathrm{m}}(\omega - \delta )} \right|^2 + (\bar n + 1)\left| {\chi _{\mathrm{m}}(\omega + \delta )} \right|^2 } \right.\\ \left. + \, {{\cal{C}}\left| {\chi _{\mathrm{m}}(\omega - \delta ) - \chi _{\mathrm{m}}(\omega + \delta )} \right|^2} \right],\end{array}$$

where $\chi _{\mathrm{m}}(\omega ) = ( - i\omega + \Gamma _{{\mathrm{eff}}}{\mathrm{/}}2)^{ - 1}$ is the mechanical susceptibility of the oscillator with total mechanical linewidth Γ_eff_, $\eta$ the overall detection efficiency, and ${\cal{C}} = 4g_0^2n_{\mathrm{p}}{\mathrm{/}}\kappa \Gamma _{{\mathrm{eff}}}$ the optomechanical cooperativity proportional to the input power. Here, *g*_0_ is the vacuum optomechanical coupling strength and *n*_p_ the mean number of intracavity photons due to each probe.

The PSD in Eq. (1) is normalized to the vacuum noise level, given by the constant 1 in the first term. The first and second terms in brackets correspond to the anti-Stokes and Stokes scattered motional sidebands, respectively, having the Lorentzian shape of the mechanical susceptibility. These exhibit the well-known motional sideband asymmetry^2,41–44,46–48^, resulting from the ratio $(\bar n + 1){\mathrm{/}}\bar n$ between absorption and emission rates. The last term in brackets is the QBA due to quantum noise in the probe light. When $\delta \gg \Gamma _{{\mathrm{eff}}}$, QBA appears as heating of the oscillator, adding $\overline n _{{\mathrm{BA}}} = {\cal{C}}$ mean quanta (Fig. 1c). The two QBA components have opposite phase. When *δ* = 0, QBA is cancelled, yielding a pristine measurement of the oscillator with PSD $\bar S_{II}(\omega ) = 1 + \eta \Gamma _{{\mathrm{eff}}}{\cal{C}}\bar S_{XX}(\omega )$ where $\bar S_{XX}(\omega ) = (\Gamma _{{\mathrm{eff}}}{\mathrm{/}}2)(2\bar n + 1)\left| {\chi _{\mathrm{m}}(\omega )} \right|^2$ is the noise spectral density of quadrature $\hat X$ (Fig. 1d). In this case, $2\overline n _{{\mathrm{BA}}}$ quanta are added to the complementary quadrature^30^ (it is noteworthy that our definition of $\overline n _{{\mathrm{BA}}}$ is half that of ref. ^30^, in order to facilitate comparison with conventional position measurement using homodyne detection). In principle, one can then increase signal-to-noise ratio (i.e., measurement sensitivity) indefinitely, with no deleterious effects on the measurement, simply by increasing probing power. In Eq. (1) we have neglected bad-cavity effects where photons scattered out of resonance interact with counter-propagating terms (neglected in the RWA) to induce QBA^30^, resulting in $\bar n_{{\mathrm{bad}}} = (\kappa {\mathrm{/}}4\Omega _m)^2{\cal{C}}$ added quanta. In our experiment, ${\cal{C}}\, \lesssim\, 10$ and thus $\bar n_{{\mathrm{bad}}}\,\, \lesssim\, 10^{ - 2}$ is completely negligible.

### Experimental setup

**Fig. 2:**
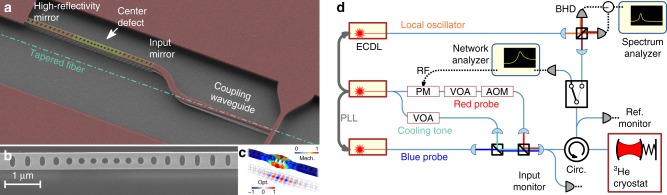
Optomechanical crystal and experimental setup. **a** False-color SEM image of the silicon optomechanical crystal cavity with a waveguide for laser input coupling. The path of the tapered fiber is indicated. **b** SEM image detail of the cavity. **c** Illustration of the mechanical breathing mode and optical mode. **d** Experimental setup. AOM, acousto-optical modulator; BHD, balanced heterodyne detector; ECDL, external-cavity diode laser; PLL, phase-locked loop; PM, phase modulator; VOA, variable optical attenuator

We performed a BAE measurement in a silicon nanobeam optomechanical crystal^49^, shown in Fig. 2a. Optically, the device functions as a single-sided cavity with a partially transmitting input mirror. Light is evanescently coupled from a tapered optical fiber into a waveguide that forms part of the nanobeam, with a coupling efficiency exceeding 50%. The optical resonance is at 1540 nm with a linewidth of $\kappa {\mathrm{/}}2\pi = 1.7\,{\mathrm{GHz}}$, of which $\kappa _{{\mathrm{ex}}} = 0.3\kappa$ are extrinsic losses to the input mirror. The optical mode is optomechanically coupled to a mechanical breathing mode of frequency Ω_m_/2*π* = 5.3 GHz, strongly confined due to a phononic bandgap, and an intrinsic linewidth of Γ_int_/2*π* = 84 kHz. This places the system in the resolved sideband regime^50^. The optomechanical coupling parameter is *g*_0_/2*π* = 780 kHz. Full details of the device design, the setup, and the system are given elsewhere^44^.

The system is placed in a ^3^He buffer-gas cryostat (Oxford Instruments HelioxTL), allowing cryogenic operation down to 0.5 K. As detailed in ref. ^44^, the buffer-gas environment allows us to overcome the prohibitive optical absorption heating in vacuo that has limited operation with these devices to very low photon numbers^51^ or pulsed operation^52–55^. We are thus able to operate at high probe powers where QBA is observable. The buffer gas causes additional damping, increasing the mechanical linewidth to Γ_m_ = Γ_int_ + Γ_gas_. In addition to the BAE probes, we also apply a cooling tone red-detuned from the optical resonance, to lower the thermal occupation $\bar n$ through optomechanical sideband cooling^50^, $\bar n \simeq \bar n_{{\mathrm{th}}}{\mathrm{/}}(1 + {\cal{C}}_{{\mathrm{cool}}})$ with $\overline n _{{\mathrm{th}}}$ the occupation of the thermal environment. The cooling tone also provides additional damping due to dynamical backaction, $\Gamma _{{\mathrm{eff}}} = \Gamma _{\mathrm{m}}(1 + {\cal{C}}_{{\mathrm{cool}}})$, which is the effective linewidth seen by the BAE probes. It is noteworthy that the probes, equal in power and oppositely detuned from cavity resonance, do not produce such dynamical backaction. Here, ${\cal{C}}_{{\mathrm{cool}}} = {\cal{C}}_0n_{\mathrm{c}}$ is the cooling tone cooperativity defined similar to ${\cal{C}}$ but relative to the original linewidth Γ_m_, with the single photon cooperativity ${\cal{C}}_0 \equiv 4g_0^2{\mathrm{/}}\kappa \Gamma _{\mathrm{m}}$, and *n*_c_ the mean intracavity photons due to the cooling tone. The cooling tone is tuned 2*π* × 220 MHz away from the red-detuned BAE probe to mitigate recently reported Kerr-type effects^44^.

The experimental setup is shown in Fig. 2d. The two BAE probes (as well as the cooling tone) are derived from two phase-locked lasers (Methods section). The three tones are combined in a free-space setup and coupled with the same polarization into a single-mode fiber. By blocking each beam path we ascertain equal power for each probe, stable to within 1%. The light reflected from the oscillator is directed to a BHD setup, where it is mixed with a local oscillator (LO) generated by a third laser. As detailed in ref. ^44^, by carefully characterizing our lasers we have determined that classical laser noise is negligible in our system. Specifically, we operate far from the relaxation-oscillation peak of our diode laser^56^. Thus, our detection is quantum-limited, as in Eq. (1). In order to accurately tune the probes across the optical resonance, we temporarily switch the reflected light to a coherent response measurement setup (Methods section).

### Observation of backaction evasion

**Fig. 3:**
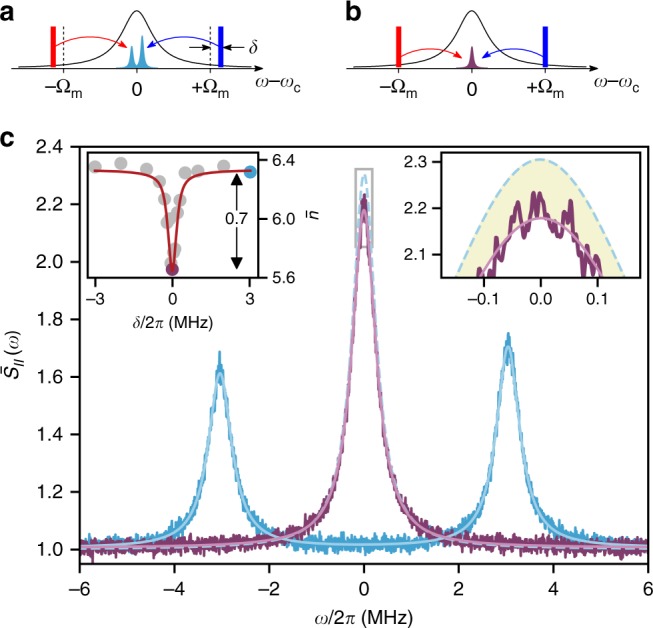
Experimental observation of backaction evasion. **a**, **b** Non-BAE and BAE measurements, respectively, as explained in the main text (see also Fig. 1c, d). **c** Data traces, normalized to the vacuum noise level, for non-BAE (cyan) and BAE measurements (purple) are shown with Lorentzian fits. The non-BAE sidebands exhibit motional asymmetry, used to self-calibrate the measurement in units of mechanical quanta. The sum of the non-BAE sidebands, indicated in dashed cyan, is larger than in the BAE case (purple) by 0.7 mechanical quanta. The right inset is a zoom of the indicated region. The left inset shows the inferred occupation $\bar n$ as a function of the detuning *δ*, with an analytic curve based on Eq. (1) with no free parameters. In this measurement, *n*_P_ = 290, *n*_c_ = 320, and ${\cal{C}}_{{\mathrm{cool}}} = 3.8$

Figure 3 shows BAE measurement of the mechanical oscillator, taken at a cryostat temperature of 2.0 K ($\overline n _{{\mathrm{th}}} \sim 7.9$) and buffer-gas pressure of 46 mbar. In this experiment, we vary the detuning *δ*/2*π* from +3 to −3 MHz. The total mechanical linewidth across the measurement is Γ_eff_/2*π* = 607 ± 7 kHz and the other measurement parameters are *n*_p_ = 290, *n*_c_ = 320, and ${\cal{C}}_{{\mathrm{cool}}} = 3.8$. When the probes are tuned away from the mechanical sidebands, *δ*/2*π* = 3 MHz, the PSD exhibits motional sideband asymmetry that can be used to self-calibrate the measurement in terms of mechanical quanta (including QBA heating; see left inset of Fig. 3c), yielding $\bar n + \bar n_{{\mathrm{BA}}} = 6.3$ in this case^42–44,48^. When tuning the probes on the mechanical sidebands, *δ*/2*π* = 0 MHz, the total thermomechanical noise is reduced by 0.7 mechanical quanta, in perfect agreement with independently calculated ${\cal{C}} = 0.7$. Thus, more than 11% of the noise in the non-BAE case is due to QBA. This constitutes the first BAE measurement in the optical domain and the first with quantum-limited detection.

We now turn to discuss technical limitations of BAE measurement imposed by our system. In conventional cavity-based position measurements employing homodyne detection^11,12^, one refers the on-resonance readout to mechanical quanta, i.e., expressing the peak of the measured PSD as $\bar S_{II}^{{\mathrm{hom}}}(\Omega_{\mathrm{m}}) \propto \bar n_{{\mathrm{imp}}}^{{\mathrm{hom}}} + \overline n _{{\mathrm{BA}}} + (\bar n + \frac{1}{2})$, where $\overline n _{{\mathrm{imp}}}^{{\mathrm{hom}}} = (16\eta {\cal{C}})^{ - 1}$ is the measurement imprecision due to shot noise (cf. Fig. 1d). The Heisenberg uncertainty relation requires $4\sqrt {\overline n _{{\mathrm{imp}}}^{{\mathrm{hom}}}\overline n _{{\mathrm{BA}}}} \ge 1$. The SQL is achieved by minimizing the total added noise $\overline n _{{\mathrm{add}}} = \overline n _{{\mathrm{imp}}}^{\hom } + \overline n _{{\mathrm{BA}}}$ subject to this constraint, yielding $\overline n _{{\mathrm{add}}}^{{\mathrm{SQL}}} = (4\eta )^{ - 1/2}$ ($\frac{1}{2}$ for ideal measurement). In a BAE measurement, there is no QBA component; however, any device suffers extraneous heating due to optical absorption, adding excess heating backaction $\overline n _{{\mathrm{BA}}}^{{\mathrm{th}}} = \beta {\cal{C}}$ analogous to $\overline n _{{\mathrm{BA}}}$. In addition, in heterodyne detection $\overline n _{{\mathrm{imp}}} = (8\eta {\cal{C}})^{ - 1}$, due to twice the vacuum noise compared with homodyne detection (“image band”). The minimum added noise is $\bar n_{{\mathrm{add}}}^{{\mathrm{th}}} = \sqrt {\beta {\mathrm{/}}2\eta }$. When $\beta \, < \, \frac{1}{2}$, BAE outperforms conventional measurement of the same efficiency.

**Fig. 4:**
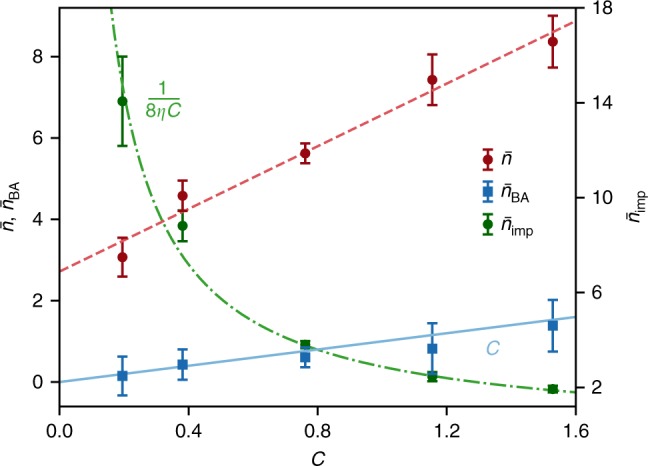
Effect of probe power on quantum backaction and optical absorption heating. The measurements were carried out at 1.6 K and ^3^He buffer-gas pressure of 30 mbar, with $n_c \simeq 420$ (${{\cal{C}}_{{\mathrm{cool}}} \simeq 5.0}$). The occupation $\bar n$ and the number of evaded quantum backaction phonons $\overline n _{{\mathrm{BA}}}$ vs. independently measured ${\cal{C}}$ are plotted on the left axis. For each value of ${\cal{C}}$, a sweep similar to Fig. 3 was performed, consisting of 7 noise spectra with *δ*/2*π* = {0, ±0.5, ±3, ±4} MHz. Each sweep was self-calibrated using motional sideband asymmetry applied to the four outermost spectra (non-backaction-evading, see Fig. 3), taking the mean and SD of the four calibrations to infer data and error bars, respectively. The solid blue line plots $\overline n _{{\mathrm{BA}}} = {\cal{C}}$. The dashed red line is a linear fit to $\bar n$ with slope $\beta {\cal{C}}$ where *β* = 3.85. The right axis shows the imprecision noise with a fit $\overline n _{{\mathrm{imp}}} = 1{\mathrm{/}}8\eta {\cal{C}}$ yielding *η* = 0.04

Figure 4 shows a set of measurements done at 1.6 K ($\overline n _{{\mathrm{th}}} \simeq 6.3$) and buffer-gas pressure of 30 mbar with variable probe power *n*_P_ and constant cooling tone power *n*_c_ = 420 (set by the maximum probe power). Both $\bar n$ and $\overline n _{{\mathrm{BA}}}$ are plotted against the independently measured cooperativity ${\cal{C}}$, with $\overline n _{{\mathrm{BA}}}$ in excellent agreement with theory (blue solid line, slope of 1). The maximum QBA cancellation reached is $\overline n _{{\mathrm{BA}}} = 1.4$ out of $\bar n + \overline n _{{\mathrm{BA}}} = 9.8$ quanta, or 14% (reduction of 0.67 dB). The linear fit to $\bar n$ yields *β* = 3.85. Thus, although QBA is evaded in our measurement, extraneous heating is still a limiting factor, as can be seen directly from Fig. 4. The imprecision noise is also in excellent agreement with theory and yields *η* = 0.04, in agreement with previous measurements of the same system^44^. Thus, $4\sqrt {\overline n _{{\mathrm{imp}}}\overline n _{{\mathrm{BA}}}^{{\mathrm{th}}}} = \sqrt {2\beta {\mathrm{/}}\eta } = 13.88$. Compared with a measurement at the SQL with the same efficiency, the optimal added noise is $\overline n _{{\mathrm{add}}}^{{\mathrm{th}}} = 2.78 \times \overline n _{{\mathrm{add}}}^{{\mathrm{SQL}}}$.

## Discussion

We have explicitly demonstrated evasion of QBA in the optical domain. Our measurements are limited by extraneous absorption heating, which currently prohibits surpassing the SQL. This highlights the challenge to overcome such effects in optical measurements. Further improvements in device design and fabrication (to be published) increased the intrinsic optical *Q*, reducing the intrinsic cavity losses. This diminishes the effect of heating, while at the same time increases the detection efficiency. This may allow reaching the performance of microwave experiments, while taking advantage of quantum-limited detection, which is easily accessible in the optical domain. For example, in the microwave domain, ref. ^33^ reported backaction evasion of 10.7 dB, but suffered from low efficiency owing to classical amplifier noise. We note that for very strong probing, extremely accurate tuning of the probes must be achieved in order to avoid the recently reported optomechanical two-tone instability^57^. Our measurement opens the path for creating motional squeezed states through reservoir engineering^34^ demonstrated so far only in the microwave domain^35–38^ and generation of squeezed light through mechanical dissipation^58,59^.

## Methods

### Theory

The theory of two-tone BAE measurements in optomechanics is already well established^30^, but we repeat the key elements here for convenience of the reader. The system is described by the Hamiltonian

2 $$\hat H = \hbar \omega _{\mathrm{c}}\hat a^\dagger \hat a + \hbar \Omega _{\mathrm{m}}\hat b^\dagger \hat b - \hbar g_0\hat a^\dagger \hat a(\hat b^\dagger + \hat b) + \hat H_{{\mathrm{drive}}}$$

where $\hat a$ and $\hat b$ are the annihilation operators of a cavity photon and a mechanical phonon, respectively. The cavity is driven by a coherent drive $\alpha _{{\mathrm{in}}}(t) = (\alpha _ + e^{ - i\Omega t} + \alpha _ - e^{i\Omega t})e^{ - i\omega _{\mathrm{l}}t}$ with carrier frequency *ω*_l_ = *ω*_c_ + Δ and amplitude-modulated at frequency Ω = Ω_m_ + *δ*, giving $\hat H_{{\mathrm{drive}}} = i\sqrt \kappa [\alpha _{{\mathrm{in}}}(t)\hat a^\dagger - \alpha _{{\mathrm{in}}}^ \ast (t)\hat a]$.

We follow standard procedure in cavity optomechanics^4^. We move to the interaction picture with respect to the Hamiltonian $\hat H_0 = \hbar \omega _{\mathrm{l}}\hat a^\dagger \hat a + \hbar \Omega \hat b^\dagger \hat b$ and linearize the operators $\hat a \to \bar a + \delta \hat a$, $\hat b \to \bar b + \delta \hat b$. In this rotating frame, we can write $\hat H = \hat H_{{\mathrm{RWA}}} + \hat H_{{\mathrm{CR}}}$ with

3 $$\hat H_{{\mathrm{RWA}}}/\hbar = - \Delta \delta \hat a^\dagger \delta \hat a - \delta \cdot \delta \hat b^\dagger \delta \hat b - [(g_ + \delta \hat b^\dagger + g_ - \delta \hat b)\delta \hat a^\dagger + (g_ + \delta \hat b + g_ - \delta \hat b^\dagger )\delta \hat a]$$

with $g_ \pm = g_0\bar a_ \pm$ the drive-enhanced coupling, where $\bar a_ \pm$ is the intracavity amplitude due to each drive tone. The counter-rotating Hamiltonian

4 $$\hat H_{{\mathrm{CR}}}{\mathrm{/}}\hbar = - [g_ + e^{ - 2i\Omega t}\delta \hat b + g_ - e^{2i\Omega t}\delta \hat b^\dagger ]\delta \hat a^\dagger - [g_ + e^{2i\Omega t}\delta \hat b^\dagger + g_ - e^{ - 2i\Omega t}\delta \hat b]\delta \hat a$$

contains off-resonant terms. Their effect has been studied perturbatively^30^ and through an exact solution^60^, but they can be neglected in resolved sideband regime $\Omega _{\mathrm{m}} \gg \kappa$, relevant for the present experiment. Including the coupling to the mechanical and optical baths, and using standard input–output theory leads to the quantum Langevin equations^61^

5 $$\delta \dot{\hat a} = - \left( {\kappa {\mathrm{/}}2 - i\Delta } \right)\delta \hat a + i\left( {g_ - \delta \hat b + g_ + \delta \hat b^\dagger } \right) + \sqrt \kappa \delta \hat a_{{\mathrm{in}}}$$

6 $$\delta \dot{\hat b} = - (\Gamma _{{\mathrm{eff}}}/2 - i\delta )\delta \hat b + i(g_ - \delta \hat a + g_ + \delta \hat a^\dagger ) + \sqrt {\Gamma _{{\mathrm{eff}}}} \delta \hat b_{{\mathrm{in}}},$$

where Γ_eff_ is the dissipation rate of the mechanical oscillator and we have introduced the optical ($\delta \hat a_{{\mathrm{in}}}$) and mechanical ($\delta \hat b_{{\mathrm{in}}}$) input noise operators. We have assumed for simplicity no intrinsic optical losses (highly overcoupled cavity). In the rotating frame, the mechanical quadrature operators are given by $\hat X = \frac{1}{{\sqrt 2 }}(e^{i\delta t}\delta \hat b^\dagger + e^{ - i\delta t}\delta \hat b)$ and $\hat Y = \frac{i}{{\sqrt 2 }}(e^{i\delta t}\delta \hat b^\dagger - e^{ - i\delta t}\delta \hat b)$. When *g*_+_ = *g*_−_ and *δ* = 0, the optical field couples exclusively $\hat X$ [Eq. (5)], the key feature of BAE measurement.

By transforming the Langevin Eqs. (5) and (6) to Fourier space, we can relate the field operators in simple matrix form ${\mathbf{d}}(\omega ) = \mathbf{\chi} (\omega ){\mathbf{L}}{\kern 1pt} {\mathbf{d}}_{{\mathrm{in}}}(\omega )$ with ${\mathbf{d}} = (\delta \hat a,\delta \hat a^\dagger ,\delta \hat b,\delta \hat b^\dagger )^T$, ${\mathbf{d}}_{{\mathrm{in}}} = (\delta \hat a_{{\mathrm{in}}},{\kern 1pt} \delta \hat a_{{\mathrm{in}}}^\dagger ,\delta \hat b_{{\mathrm{in}}},{\kern 1pt} \delta \hat b_{{\mathrm{in}}}^\dagger )^T$, ${\mathbf{L}} = {\mathrm{diag}}(\sqrt \kappa ,\sqrt \kappa ,\sqrt {\Gamma _{{\mathrm{eff}}}} ,\sqrt {\Gamma _{{\mathrm{eff}}}} )$, and

7 $${\mathbf{\chi}} (\omega ) = \left( {\begin{array}{*{20}{c}} {\chi _c^{ - 1}(\omega + \Delta )} & 0 & { - ig_ - } & { - ig_ + } \\ 0 & {\chi _c^{ - 1}(\omega - \Delta )} & {ig_ + } & {ig_ - } \\ { - ig_ - } & { - ig_ + } & {\chi _m^{ - 1}(\omega + \delta )} & 0 \\ {ig_ + } & {ig_ - } & 0 & {\chi _m^{ - 1}(\omega - \delta )} \end{array}} \right)^{ - 1}$$

with $\chi_c(\omega)=(-i\omega+\kappa/2)^{-1}$ and $\chi_m(\omega)=(-i\omega+\Gamma_{\mathrm{eff}}/2)^{-1}$. The output fields are given by the input–output relations, e.g., $\delta \hat a_{{\mathrm{out}}} = \delta \hat a_{{\mathrm{in}}} - \sqrt \kappa \delta \hat a$, yielding the matrix equation ${\mathbf{d}}_{{\mathrm{out}}} = [1 - {\mathbf{L}}{\kern 1pt} {\mathbf{\chi (\omega )}}{\kern 1pt} {\mathbf{L}}]{\mathbf{d}}_{{\mathrm{in}}}$, with ${\mathbf{d}}_{{\mathrm{out}}} = (\delta \hat a_{{\mathrm{out}}},\delta \hat a_{{\mathrm{out}}}^\dagger ,\delta \hat b_{{\mathrm{out}}},\delta \hat b_{{\mathrm{out}}}^\dagger )^T$.

The output optical field is detected using BHD, mixing it with a strong LO with frequency *ω*_l_ + Δ_LO_ (in the lab frame) on a beamsplitter and subtracting the detected intensity from the two beamsplitter output arms. This yields photocurrent with symmetrized PSD^62^

8 $$\bar S_{II}(\omega ) \propto S_{\delta \hat a_{{\mathrm{out}}}\delta \hat a_{{\mathrm{out}}}}(\Delta _{{\mathrm{LO}}} + \omega ) + S_{\delta \hat a_{{\mathrm{out}}}^\dagger \delta \hat a_{{\mathrm{out}}}^\dagger }(\Delta _{{\mathrm{LO}}} - \omega ).$$

Using the solutions for $\delta \hat a_{{\mathrm{out}}}$, $\delta \hat a_{{\mathrm{out}}}^\dagger$, and the correlations of the input noise operators

9 $$\langle \delta \hat a_{{\mathrm{in}}}^\dagger (\omega )\delta \hat a_{{\mathrm{in}}}(\omega {\prime})\rangle = 0$$

10 $$\langle \delta \hat a_{{\mathrm{in}}}(\omega )\delta \hat a_{{\mathrm{in}}}^\dagger (\omega {\prime})\rangle = \delta (\omega + \omega {\prime})$$

11 $$\langle \delta \hat b_{{\mathrm{in}}}^\dagger (\omega )\delta \hat b_{{\mathrm{in}}}(\omega {\prime})\rangle = \bar n\delta (\omega + \omega {\prime})$$

12 $$\langle \delta \hat b_{{\mathrm{in}}}(\omega )\delta \hat b_{{\mathrm{in}}}^\dagger (\omega {\prime})\rangle = (\bar n + 1)\delta (\omega + \omega {\prime})$$

where $\bar n$ denotes the mean thermal occupation of the environment seen by the oscillator, the photocurrent PSD (8) can be evaluated. Apart from a white noise floor due to shot noise (10), both PSDs $S_{\delta \hat a_{{\mathrm{out}}}\delta \hat a_{{\mathrm{out}}}}(\omega )$ and $S_{\delta \hat a_{{\mathrm{out}}}^\dagger \delta \hat a_{{\mathrm{out}}}^\dagger }(\omega )$ contain information near $\omega \approx \Gamma _{{\mathrm{eff}}},\delta \ll \Delta _{{\mathrm{LO}}}$, and appear shifted by Δ_LO_ in the photocurrect PSD (8). We refer the measured PSD to Δ_LO_ by setting Δ_LO_ = 0 (see Fig. 1c, d).

We now specialize to the case Δ = 0 and *g*_+_ = *g*_−_, as in our experiment. We can also approximate $\chi _{\mathrm{c}}(\omega ) \approx \chi _{\mathrm{c}}(0)$, as for our frequencies of interest $\omega \ll \kappa$. Including finite detection efficiency *η* finally yields the PSD given in the main text, normalized to the vacuum noise level,

13 $${\bar S_{II}(\omega ) = 1 + \eta \Gamma _{{\mathrm{eff}}}^2{\cal{C}}\left[ {\bar n\left| {\chi _{\mathrm{m}}(\omega - \delta )} \right|^2 + \, (\bar n + 1)\left| {\chi _{\mathrm{m}}(\omega + \delta )} \right|^2 + \, {\cal{C}}\left| {\chi _{\mathrm{m}}(\omega - \delta ) - \chi _{\mathrm{m}}(\omega + \delta )} \right|^2} \right]}$$

with optomechanical cooperativity ${\cal{C}} = 4g_0^2n_{\mathrm{p}}{\mathrm{/}}\kappa \Gamma _{{\mathrm{eff}}}$ and $n_{\mathrm{p}} = |\bar a_\pm|^2$. For *δ* = 0, the quadrature $\hat X$ is given by $\hat X(\omega) = \sqrt {\Gamma _{{\mathrm{eff}}}/2} \chi _{\mathrm{m}}(\omega )[b_{{\mathrm{in}}}^\dagger (\omega ) + b_{{\mathrm{in}}}(\omega )]$ with PSD $\bar S_{XX}(\omega ) = (\Gamma _{{\mathrm{eff}}}/2)(2\bar n + 1)\left| {\chi _{\mathrm{m}}(\omega )} \right|^2$.

### Frequency setup and phase lock

**Fig. 5:**
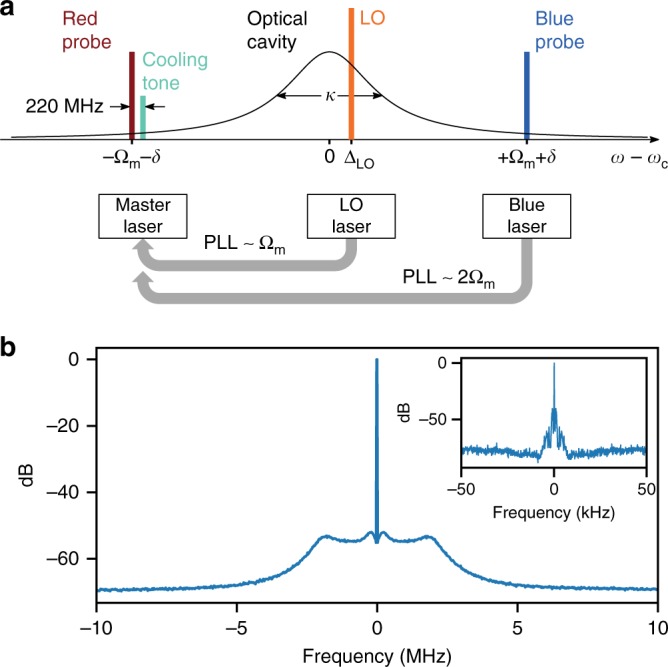
Complete frequency setup and phase-locked loop beat note. **a** The frequencies of the various tones used in the experiment. The cavity has resonance frequency *ω*_c_ and linewidth *κ*. The mechanical resonance frequency is Ω_m_. The probes are applied with small detuning *δ* from the upper and lower mechanical sidebands. An additional cooling tone is applied as shown. The local oscillator (LO), which does not enter the cavity, is detuned by Δ_LO_ from cavity resonance. **b** A typical out-of-loop PLL beat note, relative to the offset frequency. The resolution bandwidth (RBW) is 6.25 kHz. The inset shows a zoom-in with RBW of 31.25 Hz

Figure 5a shows the complete picture of the various laser tones applied in the experiment. A master laser generates the red-detuned probe and, via an acousto-optic frequency shifter, the cooling tone. Two other lasers are referenced to the master laser through a phased-locked loop and are locked at a higher frequency. One laser, locked at an offset ~ Ω_m_, generates the LO for the BHD. The second laser, locked at an offset ~ 2Ω_m_, generates the blue-detuned probe.

We perform the phase-lock using a PID controller with 10 MHz bandwidth to control the current of the diode lasers. A typical beat note is shown in Fig. 5b. The residual phase error^63–65^, computed from the ratio coherent to total power, is $\langle \sigma _\varphi ^2\rangle \simeq 5 \times 10^{ - 3} \,\, {\mathrm{rad}}^2$, limited by the resolution bandwidth.

### Optomechanical cooling and absorption heating

At low temperatures, intracavity photons shift the optical resonance to higher frequencies, due to a combination of thermo-optic and thermal expansion effects in silicon. Excess blue-detuned pumping can cause thermal run-off, making operation unstable when the cavity is driven with the BAE probes alone (due to the presence of the blue-detuned probe). An additional red-detuned (cooling) tone of sufficient power is required. With the sample used in this experiment, we have found empirically that we need $n_{\mathrm{c}} \,\gtrsim \, n_{\mathrm{p}}{\mathrm{/}}2$ for stable operation, where *n*_c_ denotes the intracavity photons due to the cooling tone and *n*_P_ denotes the intracavity photons due to each probe.

### Probe tuning via coherent response

**Fig. 6:**
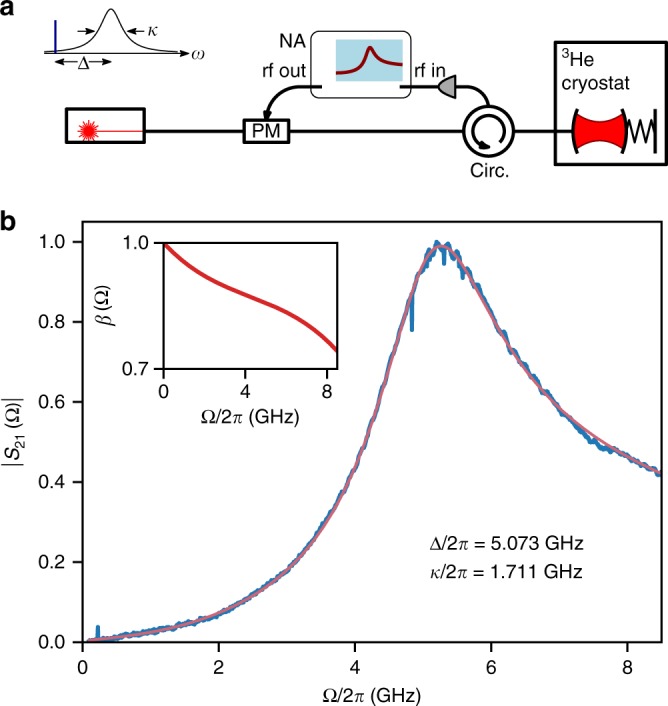
Coherent response determination of detuning and linewidth. **a** Simplified setup for optical measurements using coherent response (part of the full experimental setup). **b** An example of a measurement with a fit Eq. (17), yielding cavity linewidth *κ* and detuning Δ. A correction due to extraneous frequency-dependent response, measured and shown in the inset, is included in the fit

In experiments such as this, it is of utmost importance to tune the probes accurately around the optical resonance. Active locking to the cavity (e.g., using a Pound–Drever–Hall technique) are inappropriate for a single-sided cavity and also result in driving the cavity on resonance. We use passive tuning of our master laser, from which the other tones are derived, using the cavity coherent response, similar to previous experiments^66^. From the response curve we extract accurate values of both Δ and *κ*. Here we give details on the method. A simplified setup is shown in Fig. 6a. The laser is phase-modulated using RF output of a network analyzer (NA). The carrier and sidebands reflected from the cavity interfere on a fast photodetector and the photocurrent fed to the NA input, measuring the magnitude of the *S*_21_ parameter. The amplitude incident on the cavity is given by

14 $$a_{{\mathrm{in}}}(t) \simeq a_0\left(1 + \frac{\beta }{2}e^{i\Omega t} - \frac{\beta }{2}e^{ - i\Omega t}\right),$$

with *β* the modulation index and the reflected light is

15 $$a_{{\mathrm{out}}}(t) \simeq a_0 \left[r(\Delta ) + \frac{\beta }{2}r(\Delta + \Omega )e^{i\Omega t} - \frac{\beta }{2}r(\Delta - \Omega )e^{ - i\Omega t}\right],$$

with

16 $$r(\Delta ) = 1 - \frac{{\eta _{\mathrm{c}}\kappa }}{{\kappa {\mathrm{/}}2 - i\Delta }}$$

the amplitude reflection coefficient at detuning Δ and $\eta _{\mathrm{c}} \equiv \kappa _{{\mathrm{ex}}}{\mathrm{/}}\kappa$ the cavity coupling parameter. The magnitude of the *S*_21_ parameter, the Ω frequency component of the photocurrent $\left| {a_{{\mathrm{out}}}(t)} \right|^2$, is given by (here and below we omit a constant scale factor)

17 $$\left| {S_{21}(\Omega )} \right| = \frac{\beta }{2}\left| {r(\Delta )r^ \ast (\Delta - \Omega ) - r^ \ast (\Delta )r(\Delta + \Omega )} \right|.$$

Figure 6b shows a typical coherent response measurement, which deviates significantly from a Lorentzian when Δ ~ *κ*, as in our case. In addition, when scanning over a wide bandwidth, one has to take into account the frequency dependence of *β* (due to phase modulator, rf cables, detector response, etc.). A robust and reliable procedure to calibrate the frequency dependence of the entire detection chain is to take several traces at various detunings and fit all of them simultaneously to Eq. (17) with only Δ variable across traces, and with a high-order polynomial in Ω as a prefactor. This prefactor is then applied in all subsequent fits. The inset in Fig. 6b shows the frequency dependence of *β* given by the polynomial. We adjust probe detuning using this method before each data point acquisition. By repeatedly acquiring Δ in a single instance, we estimate our accuracy to be ±20 MHz (±0.01 *κ*).

## Data Availability

The code and data used to produce the plots within this paper are available at 10.5281/zenodo.2563797. All other data used in this study are available from the corresponding authors upon reasonable request.
